# Differentially Expressed Proteins in Intra Synovial Compared to Extra Synovial Flexor Tendon Grafts in a Rabbit Tendon Transplantation Model

**DOI:** 10.3390/biomedicines8100408

**Published:** 2020-10-12

**Authors:** Simon Farnebo, Monica Wiig, Björn Holm, Bijar Ghafouri

**Affiliations:** 1 Department of Hand Surgery, Plastic Surgery, and Burns, Linköping University, SE 581 83 Linköping, Sweden; 2 Department of Surgical Science, Hand Surgery, Uppsala University, and Uppsala University Hospital, 751 85 Uppsala, Sweden; monica.wiig@gmail.com (M.W.); Bjorn.Holm@surgsci.uu.se (B.H.); 3 Pain and Rehabilitation Centre, and Department of Health, Medicine and Caring Sciences, Linköping University, SE 581 83 Linköping, Sweden

**Keywords:** hand surgery, tendon adhesion, biomarkers, extra synovial graft, intra synovial graft

## Abstract

Uncomplicated healing of grafts for tendon reconstruction remains an unsolved problem in hand surgery. Results are limited by adhesion formation and decreased strength properties, especially within the tight fibro-osseous sheath of the digits. This is especially problematic when an extra synovial tendon graft is used to replace an intra synovial flexor tendon. Compositional differences are likely to play an important role in these processes. The aim of this study was, therefore, to compare protein expression in pair-matched intra synovial tendon grafts with extra synovial tendon grafts, using a rabbit tendon injury model. We hypothesized that there would be significant differences in proteins critical for response to tensile loading and adhesion formation between the two groups. Using mass spectrometry and multivariate statistical data analysis, we found tissue-specific differences in 22 proteins, where 7 explained 93% (R2) of the variation, with a prediction of 81% (Q2). Among the highest discriminating proteins were Galectin, Histone H2A, and Periostin, which were found in a substantially larger amount in the extra synovial tendons compared to the intra synovial tendons. These findings may contribute to improved understanding of the differences in outcome seen after tendon reconstruction using tendon grafts with intra synovial and extra synovial grafts.

## 1. Introduction

When the flexor tendons in the hand are severely damaged by injury or degenerative disease, reconstruction with a tendon graft may be inevitable. Today only autologous grafts are available for this, even though recent improvements in the field of tissue engineering have provided promising results with decellularized tendons from donors [1]. Generally, two types of autologous grafts are available; extra synovial grafts from either the wrist or foot (palmaris longus and plantaris) or, more uncommon, intra synovial grafts from a neighboring finger. Although there are theoretical benefits with replacing a severed intra synovial tendon with an intra synovial tendon graft, this is generally considered unacceptable because of the donor morbidity to the donor finger. On the other hand, adhesion formation around the intra synovial graft is considered less than around an extra synovial tendon graft. This difference is believed to be associated with a superior surface durability of the intra synovial tendons compared to that of the extra synovial tendons. The intra synovial tendon surface is, at least, before transplantation, covered with a thin lubricating layer that consists of mainly Hyaluronic acid and lubricin, along with other phospholipids. These compounds are believed to reduce tendon gliding resistance. Extra synovial tendon grafts in canine models has also been demonstrated to undergo extensive cellular death and rapid remodeling associated with ingrowth of fibrovascular scar from peripheral tissue. This remodeling process in extra synovial tendon grafts is believed to be a key process in scar formation that is not observed to the same extent in intra synovial autografts, where cell necrosis and matrix turnover is minimal [2,3,4]. In addition, intra synovial tendons and extra synovial tendons reveal differences in expression of mRNA levels for matrix molecules and transforming growth factor-beta (TGFB1) [5], which, in turn, may affect tendon scarring.

Specific knowledge about the proteomic profile of tendons [6] is scarce and non-existent when it comes to protein changes after tendon grafting. Proteomic studies on tendons have, to some extent, focused on regulatory molecules in early tendon formation and aging [7]. These studies have shed some light on how altered protein profiles may affect the increased injury risk with increasing age [8], but data on the differences in different tendon graft types are still lacking.

Since there have been concerns regarding the suitability of using extra synovial tendon grafts in an intra synovial location [3], it is reasonable to consider that a mismatch in protein composition of the tendons may affect healing and tissue reactions, especially when an extra synovial tendon is used in an intra synovial environment.

Proteomic studies have the potential to significantly improve our knowledge of how the grafts differ after transplantation. Alterations on a protein level may, hence, shed light on the complex interplay between various proteins responsible for inflammation, structural and metabolic orchestration after transplantation, and will, hopefully, open up for mechanism-based alterations that can aid in future treatment approaches to aid patients with these highly disabling injuries. The aim of this study was, therefore, to compare protein expression in tendon grafts from a rabbit tendon injury model, comparing pair-matched intra synovial tendon grafts with extra synovial tendon grafts. We hypothesized that there would be differences in key structural components between intra synovial and extra synovial grafts, and proteins critical for the response to tensile loading, and the response to injury and concurrent adhesion formation would demonstrate significant differences between the two types of grafts. We, therefore, used a multivariate statistical model to test the null hypothesis stating that there would be no significant difference between the protein expression of the two groups.

## 2. Experimental Section

### 2.1. Surgical Procedures

Seven female young-adult New Zealand White rabbits weighing 3 kg (±0.3 kg) were used for the study. They were housed and cared for in accordance with regulations for the protection of laboratory animals. The study was approved by the Uppsala animal ethics committee (Permit Number: C 48/14, Uppsala, Sweden). 

**Figure 1:**
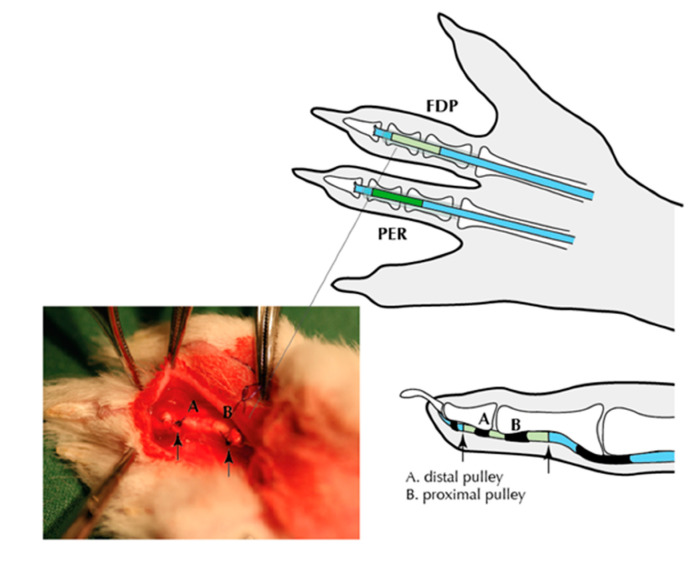
Schematic illustration of the rabbit transplant model. Flexor digitorium profundus (FDP) tendons were transferred from the neighboring digit, and Peroneus tendon (PER) tendons were transferred from a separate incision on the foreleg.

The surgical procedures were performed during anesthesia with fentanyl–fluanisone (0.3 mL/kg body weight; Hypnorm, Janssen, Beerse, Belgium) and midazolam (2 mg/kg body weight; Dormicum, Roche, Basel, Switzerland) under sterile conditions and all efforts were made to minimize suffering. A prophylactic intravenous dose of the antibiotic cefuroxime (100 mg; Zinacef, GlaxoSmithKline, London, UK) was given before surgery. Buprenorfin (0.1 mg/kg body weight; Temgesic, RB Pharmaceuticals, Berkshire, UK) was given postoperatively to prevent pain. The surgical procedures were performed on both hind paws. To decrease the tensile load on the flexor tendon’s phalangeal section, a partial division of the tendons was performed at the tendon–muscle transition. The third digit was longitudinally incised, and the flexor tendon sheath opened approximately 12 mm between the first and second annular pulley. The superficial flexor tendon was resected, and the intermediate segment of the deep flexor tendon (Flexor digitorium profundus—FDP), approximately 8 mm, between the first and second annular pulleys, was resected and transplanted to the deep flexor tendon on the third digit on the other foot (Figure 1). An extra synovial peroneus tendon (Peroneus tendon—PER), harvested from a separate incision on the foreleg, was transplanted to the deep flexor tendon on the first toe. The transplanted tendons were sutured end-to-end using a modified Kessler suture technique (5-0 Prolene, Ethicon, Sollentuna, Sweden) for the core suture, and then peripheral circumferential running sutures (6-0, PDS, Ethicon). The rabbits were sacrificed on day 28 postoperatively, and the transplanted tendons segments from both feet were harvested for further analysis, as previously described [5]. The samples were cleaned from sutures and rinsed in physiological saline. The samples were put into tared cryovials, immediately placed in liquid nitrogen, and stored at −80 °C until further analysis.

### 2.2. Protein Extraction and Digestion

The frozen tissue samples were heat stabilized with Denator Stabilizer T1 (Denator, Gothenburg, Sweden), placed in a tube containing urea sample buffer solution, homogenized by sonication, incubated for 2 h at 4 °C, followed by 1 h centrifugation at 20,000× *g* as previously described [9]. The supernatant collected and protein concentration was measured using a 2-D Quant Kit (GE Healthcare), according to the manufacture’s recommendation. The proteins were reduced by incubating in 25 mM dithiothreitol for 15 min and alkylated 75 mM iodoacetamide for an additional 15 min. The samples were diluted 8× with 25 mM ammonium bicarbonate and filtered by 3 kDa Amicon spin-filter before digestion with trypsin (1:25, *w*/*w* trypsin/protein) [10]. The digested peptides were dried in a speed vacuum concentrator, reconstituted in 0.1% of formic acid in MilliQ water, and subjected to liquid chromatography tandem mass spectrometry (LC-MS/MS) analysis.

### 2.3. LC-MS/MS Analysis

Reversed-phase chromatography was performed using the EASY-nano LC II, as described previously [10]. The injection volume was 4 μL; mobile phases consisted of 0.1% formic acid in water (A) and 0.1% formic acid in acetonitrile (B). Zero point two five micrograms of peptides were injected and separated by a two-step gradient of 2–30% B in 70 min, 30–100% B in 50 min with a flow rate of 300 nL/min. The LC was coupled to an LTQ Orbitrap Velos Pro hybrid mass spectrometer (Thermo Scientific) with a nano-electrospray source. The LTQ Orbitrap Velos Pro was operated in the data-dependent mode with survey scans acquired at a resolution of 30,000 at *m*/*z* 400.

### 2.4. Database Searches and Data Evaluation

Raw files were searched using Sequest HT in Proteome Discoverer (ThermoFisher Scientific, San Jose, CA, USA; version 1.4.0.288) using the following parameters as described previously [10]: semi trypsin was used as digestion enzyme; maximum number of missed cleavages 2; fragment ion mass tolerance 0.60 Da; parent ion mass tolerance 10.0 ppm; fixed modification–carbamidomethylation of cysteine; variable modifications–terminal acetylation, methionine oxidation. Data were filtered at a 1% false discovery rate, high peptide confidence, rank 1 peptides in top-scored proteins. The Uniprot Rabbit database available at the UniProtKB website (https://www.uniprot.org/taxonomy/9986) was used as a protein sequence database.

Identified proteins were further analyzed with SCAFFOLD software (version 4.7.3; Proteome Software Inc., Portland, OR, USA). Identifications were based on 95% peptide identification probability and 99% protein identification probability. Proteins containing identical peptides, and which could not be differentiated based on MS/MS analysis alone were grouped to satisfy the principles of parsimony. The label-free quantitative analysis of peptides was performed by spectral counting analysis using normalized exponentially modified protein abundance index (emPAI) calculated for each protein to normalize run-to-run variations.

String (Search Tool for the Retrieval of Interacting Genes/Proteins, version 10) was used for bioinformatics analysis, and proteins were categorized according to gene ontology terms [11]. The parameters; organism: Homo sapiens, maximum number of interactions: query proteins only, minimum required interaction score: medium confidence (0.400), and a false discovery rate (FDR) ≤ 0.05 was used when classifying the biological process.

### 2.5. Statistics

The data sets obtained by means of proteomic studies are complex. Using advanced, multivariate statistical analysis, these types of complex data can be analyzed. To investigate the multivariate correlation and between the membership of groups and quantified proteins, orthogonal partial least square discriminant analysis (OPLS-DA) using SIMCA-P+ version 13.0 (UMETRICS, Umeå, Sweden) was used. Before this analysis, principal component analysis (PCA) was used to check for multivariate outliers. The procedure to compute multivariate correlation models has been described earlier [9] and is in accordance with Wheelock and Wheelock [12]. The univariate statistic was performed using the statistical packages IBM SPSS Statistics (version 24.0; IBM Corporation, Route 100 Somers, New York, NY, USA). The Mann–Whitney test was performed for the comparation of the important proteins discriminating the group separation. A probability of <0.05 (two-tailed) was accepted as the criterium for significance.

## 3. Results

**Figure 2:**
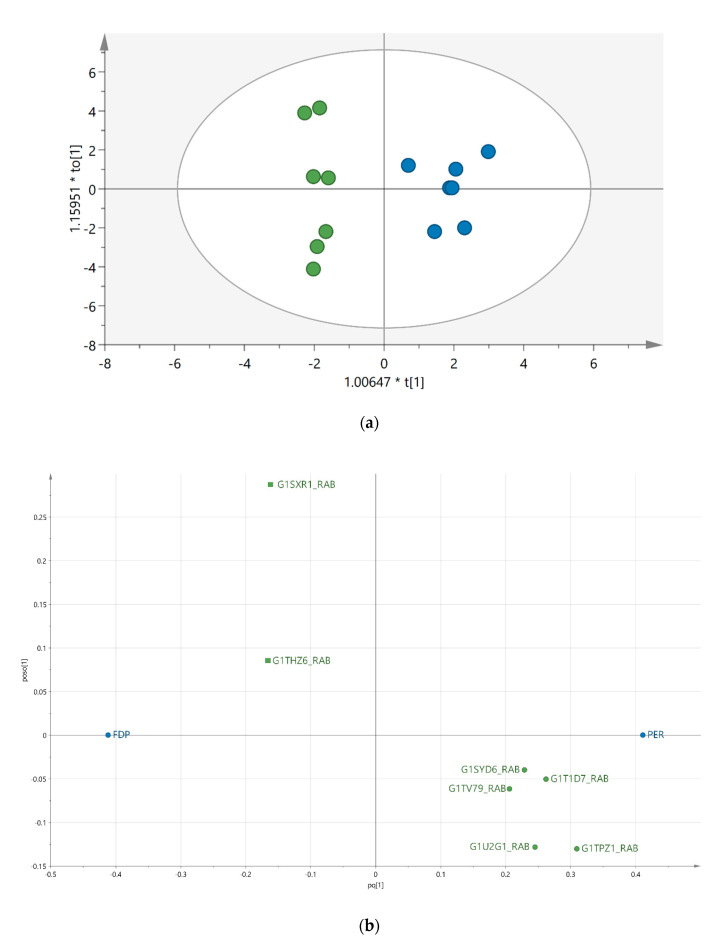
(**a**) An OPLS-DA (orthogonal partial least square discriminant analysis) model showing the discriminant separation between FDP (green filled circles and PER (blue filled circles). The longitudinal dimension (*Y*-axis) shows the interclass discrimination, and the latitudinal dimension (*X*-axis) shows the intraclass discrimination between the groups. (**b**) Loading plot corresponding to significant proteins, with a variable of importance (VIP) value > 1 important for FDP (square) and PER (circle). G1TPZ1_RABIT (Galectin), G1T1D7_RABIT (Periostin), G1U2G1_RABIT (Histone H2A), G1SYD6_RABIT (Lamin-A/C), G1TV79_RABIT (SerpinH1), G1SXR1_RABIT (Prolargin), G1THZ6_RABIT (Ig gamma chain C region).

**Figure 3:**
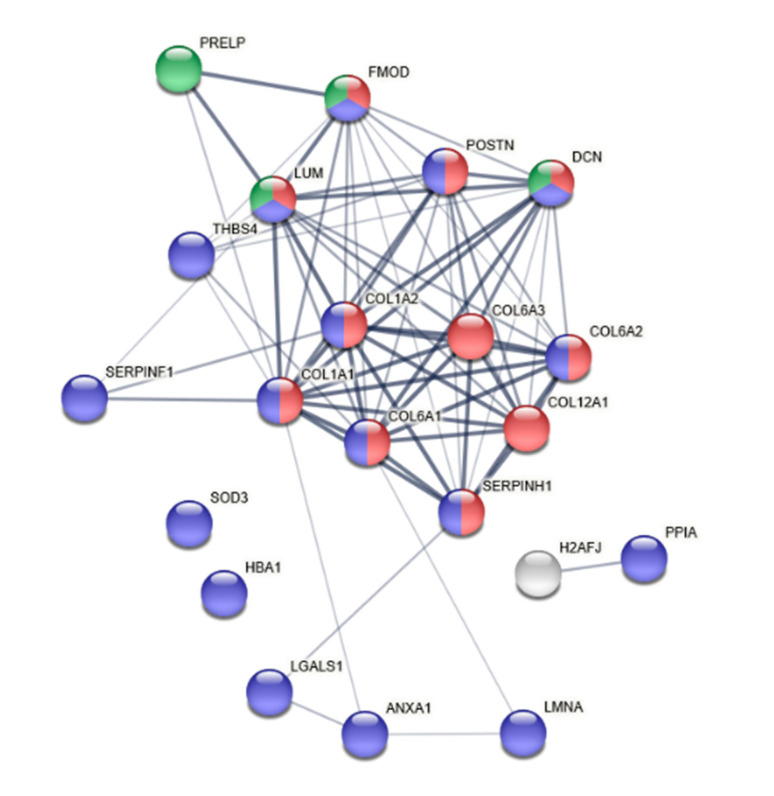
Pathway analysis of altered proteins between FDP and PER. Each protein is represented by a colored node, and protein–protein interaction is represented by an edge visualized as a line. Higher combined confidence scores are represented by thicker lines/edges. The significant protein–protein interactions enrichment (PPI) (*p*-value < 1.0 × 10^−16^) identified three biological processes that the proteins were involved in; extracellular matrix organization (red), response to chemical (blue), and glycosaminoglycan catabolic process (green).

We were able to identify in total about 600 proteins from the tissue samples. The identified proteins were classified into their biological processes, cellular components, and molecular functions according to the gene ontology analysis performed in STRING (Supplementary Materials Table S1). The analysis revealed a group of proteins (FDR < 0.05) correlated to wound healing, response to stimuli, collagen biosynthetic processes, and collagen fibril organization. One hundred and thirty proteins whose expression could be identified in at least 50% of a group were subjected to the multivariate data analysis. To identify the proteins important for group separation of the FDP and PER, an OPLS-DA regression model was generated (Figure 2a). A total of 22 proteins were considered important for the group separation (Table 1). Those proteins together explained 93% (R2) of the variation and with a prediction of 81% (Q2). The CV-ANOVA revealed that the model was highly significant (*p* = 0.008781). Among the highest discriminating proteins VIP (variable of importance) >1.2 were Galectin (G1TPZ1_RABIT), Periostin (G1T1D7_RABIT), and Histon H2A (G1U2G1_RABIT), which all were more abundant in PER compared to FDP (Figure 2b). These differences were furthermore found to be significant *p* < 0.05 (Mann–Whitney) independently of other factors, indicating that these factors constitute some of the key differences seen between PER and FDP-tendons 28 days after surgery. The protein candidates in Table 1 were subjected to STRING analysis to identify activated protein networks. The analysis revealed significant protein–protein interactions enrichment (PPI) with a *p*-value of <1.0 × 10^−16^, indicating that the proteins were at least partially biologically connected (Figure 3). The PPI grouped the identified as extracellular matrix organization (red, FDR = 4.11 × 10^−12^), response to chemical (blue, FDR = 1.72 × 10^−6^), and glycosaminoglycan catabolic process (green, FDR = 5.49 × 10^−5^). 

## 4. Discussion

Significant quantitative differences were identified in the proteome of two entities of tendon material, differentiated by whether they originally resided within or outside the fibro-osseous tendon sheath of the digits (Figure 2, Table 1). This clear separation of proteins may hold important clues to the orchestration of events that occur after tendon reconstruction using tendon grafts with intra synovial and extra synovial grafts. This is important, as in hand surgery, uncomplicated healing of autologous grafts for tendon reconstruction remains a basic, unsolved problem. Even with improved repair techniques and early active motion therapy, results are limited by adhesions and decreased strength properties after surgery. Adhesions within the tight fibro-osseous sheath in the digits cause decreased postoperative range of motion, especially, and ultimately poor hand function. Tendon adhesions are even more prevalent when an autologous extra synovial tendon graft, such as the palmaris longus or the plantaris tendon, is used to replace an intra synovial flexor tendon. Clinical and experimental studies of repair with extra synovial tendons have shown restricted tendon gliding and reduced digital function [13,14].

Autologous tendon grafting is today mostly done using extra synovial tendon grafts. These tendons, however, lack the loose connective tissue lined with synovial cells that intra synovial tendons have. In addition, our results indicate that extra synovial tendons have a different extracellular matrix (ECM) composition compared to intra synovial grafts after 28 days. The results also indicate that there are significant differences when intra synovial and extra synovial tendons are used in the reconstruction. This difference may be of importance in the processes that cause impaired function postoperatively and may thus hold keys to future treatment approaches.

Tendons consist of a systematic and densely packed organization of connective tissue. Collagen is by far the most abundant protein, and it is organized into fibrils, fibers, fiber bundles that form the ultrastructure that enables it to withstand high tensile forces. Other smaller extracellular matrix (ECM) proteins are likely to give the specific fingerprint that tendons vary markedly in design, most likely coupled to their function [6]. The nature of the individual components forms the specific properties of the tendon is equipped to withstand high tensile forces and act appropriately after injury [15]. Higher concentrations of specific proteins may, therefore, coincide with increasing risks for adhesion formation, and vice versa. 

Galectine and Periostin [16,17] have previously been identified as matricellular proteins [18]. Matricellular proteins are characterized by being dynamically expressed non-structural proteins that are present in the ECM, and may thus have implications for both tendon strength and adhesion formation.

More specifically, Galectins are a large family with relatively broad specificity, having a broad variety of functions, including mediation of cell–cell interactions, cell–matrix adhesions, and transmembrane signaling. Their role in tendon healing and repair has, however, not been studied further.

Periostin was first identified as an osteoblast-specific factor that has been shown to promote adhesion in various tissue, including bone healing. This process is mediated through increased interaction with cell surface-bound integrins [19,20,21] that are transmembrane receptors, which facilitate cell–ECM adhesion. Periostin has also been found to be involved in many other tissues and pathologies, where it primarily seems to be involved in various fibrotic conditions, including sub-epithelial fibrosis in bronchial asthma [22] as well as in bone marrow fibrosis [23]. Similar effects have been shown in flexor tendons, and it is believed that these effects are a downstream function of TNFα release, secondary to an increased inflammatory activity [24] Increased pro-inflammatory cytokines (IL-1β and TNFα) have previously been shown both after flexor tendon surgery, as well as in models of high repetition, high force forelimb training [24], with ensuing upregulation of Periostin. Altogether, it is possible that upregulation of Periostin, secondary to an increased inflammatory response after tendon reconstruction, plays a role in the increased risk for adhesion formation seen in extra synovial tendons, such as PER. One can further speculate that there is a stronger inflammatory response directed towards the extra synovial tendons compared to the intra synovial tendons. This response may, in turn, be responsible for ensuing tissue adhesion through recruitment of synovial fibroblasts as well as bone marrow derived cells [25] to the graft site.

High levels of Histone 2A was also found to be associated with the PER group. Beside the classical role to package and order the DNA into structural units, extracellular histones bind to receptors and trigger activation of multiple signaling pathways. Histone levels are also known to be significantly elevated in response to injury and are involved in the regulation of inflammation [26]. Histones have, furthermore, previously been used as an indicator of replicative damage in tendon fibroblast monolayer cultures. Altogether, it may be speculated that the increased levels of Histone 2A may be associated with an increased inflammatory reaction surrounding the extra synovial tendon grafts.

Other proteins that were more prominent in PER compared to FDP were Lamin and Serpin H1. Similarly, Prolargin and an IgG-C protein with high resemblance to Tenascin were more abundant in FDP compared to PER. In addition, as predicted, both collagen alpha-2(I) (COL1A2) and collagen alpha-3(VI) (COL6A3) were found at a higher degree in the FDP compared to the PER tendons. 

Lamins (LMNAs) are nuclear soluble proteins, localized both at the nuclear lamina and in the nuclear matrix, that are involved in the structural and functional integrity of the cell nucleus. Defects in Lamin-A maturation are, for example, found in very rare premature aging syndromes, such as Hutchinson–Gilford–Progeria-Syndrome (Progeria) [27]. Interestingly, an accumulation of LMNA in coronary endothelial cell culture supernatant has been shown to increase oxidative stress and inflammation. It is speculated that these effects are, at least to part, related to the relative toxicity of LMNA [28]. It is further speculated that increases in steady-state levels of LMNA will result in local cell dysfunction and ultimately lead to the development of age onset-pathologies, which may also have an impact on tendon healing. Clearly, the precise cause–effect relationship between the increased levels of LMNA seen in PER compared to FDP and increased adhesion formation around PER tendons requires further investigation.

Serpin H1 [29], which is more abundant in PER tendons, is also known as Heat Shock Protein 47 (Hsp47). It functions as a collagen-binding stress protein during the biosynthesis and secretion of procollagen, which, in turn, is a precursor of the main structural protein of tendons, collagen 1. The link to adhesion formation in tendons is strong as previous papers show that TGF-beta1 delays the degradation of Hsp47 mRNA via translocation of the Hsp47 gene. TGF-beta1 is a well-known promotor for peritendinous adhesion formation [30] through a specific upregulation within the tendon but not the sheath. TGF-β1 has further been shown in several different cell types to cause the upregulation of a number of matrix metalloproteinases (MMPs), namely MMP-2, and MMP-9, that also are well-known promotors for tendon adhesions, although not specifically prominent in this material. Altogether, it is possible that the adhesion effects seen on the PER tendons may be related to a TGF-β1 driven orchestration of protein activation, including both procollagen and Hsp47 genes.

Prolargin is a leucine-rich repeat protein present in the connective tissue extracellular matrix. It has been shown to function as a molecule anchoring the basement membrane to the underlying connective tissue, specifically by binding type I collagen to basement membranes. Its role in tendon healing and tendon adhesion formation has, however, not been studied previously.

Tenascin is characteristically expressed in the musculoskeletal tissues, at sites where increased mechanical tension is applied [31]. Tenascin is found in abundance in tendons, both peri cellularly and around the collagen fiber bundles, and has been shown to be drastically upregulated upon injury. It has, furthermore, been postulated that the tenocytes respond to stress by producing Tenascin to increase the elasticity of the ECM surrounding them [32] and, through its effects, have anti-adhesive properties [33,34] at healing through blocking cell interactions with fibronectins and other matrix molecules [35]. The increased levels of Tenascin, and connected EGF-like and Fibronectin type-III domains in intra synovial tendon grafts compared to extra synovial grafts, may, therefore, play a role in anti-adhesive functions seen in intra synovial flexor tendon grafting.

This study has limitations. First, the results represent a snapshot of all proteomic events that occur after tendon transplantation. To further elucidate factors and events within the proteome that affects tendon healing, prolonged and more thorough proteomic assessment over several time points must be conducted. This process is, however, quite costly, both in terms of lab work but also for the sacrifice of more animals. The statistical method used found the proteins that discern extra synovial tendons from intra synovial tendons. This method did not, however, consider whether these proteins affect tendon gliding or not, but rather separates the two groups statistically. This pilot proteomic study was a hypothesis-generating study showing that there are potential protein biomarkers involved in inflammation, which might be a good candidate for future studies investigating the healing process after a tendon reconstruction. The sample preparation was optimized for proteomic study that was not compatible with histological studies of the grafts to show the inflammatory sites and presence of main proteins Galectin and Periostin.

Future studies will be directed towards proteomic assessment of more tenogenic and adhesion specific proteins, after reconstruction. It is likely that a better understanding of the complex orchestration of tendon healing on a protein level can be used to tailor future treatment approaches in these patients. In particular, novel treatments, such as surface augmentation with lubricin and PXL-01 [36] and similar compounds, may hold key functions in decreasing the negative inflammatory reactions within the tendon sheath, inducing scar formation and a poor rehabilitation result.

## Figures and Tables

**Table 1:** Altered proteins in intra synovial Flexor digitorium profundus (FDP) and extra synovial Peroneus tendon (PER) tendon grafts. ↑= Upregulated; ↓= Downregulated in FDP compared to PER. The accession number and molecular function are based on the UniProt database (http://web.expasy.org). * refers to human ortholog. Proteins with VIP (variable influence of projection) value > 1 are considered to be important/significant for separation between FDP and PER.

Accession Number	Protein Name	Gene	Molecular Function	VIP	Alterations FDP vs. PER
G1TPZ1_RABIT	Galectin	LGALS1	Carbohydrate binding	1.54	↓
G1T1D7_RABIT	Periostin	POSTN	Heparin binding	1.25	↓
G1U2G1_RABIT	Histone H2A	H2AFJ	DNA binding	1.22	↓
G1SYD6_RABIT	Lamin-A/C	LMNA	Structural molecule activity	1.09	↓
G1TV79_RABIT	Uncharacterized protein (96.4% identity to human Serpin H1)	SERPINH1 *	Collagen-binding protein	1.06	↓
G1SXR1_RABIT	Prolargin	PRELP	Heparin binding	1.05	↑
G1THZ6_RABIT	Uncharacterized protein (84% identity to Ig gamma chain C region rabbit)	IGHG1 *IGHG2 *	Antigen binding	1.02	↑
G1T2Z5_RABIT	Collagen alpha-2(I) chain	COL1A2	Extracellular matrix structural constituent	0.98	↑
G1ST52_RABIT	Collagen alpha-3(VI) chain *	COL6A3 *	Extracellular matrix structural constituent	0.98	↑
PGS2_RABIT	Decorin	DCN	Collagen binding	0.96	↑
G1SP97_RABIT	Lumican	LUM	Collagen binding	0.96	↓
G1SWS6_RABIT	Fibromodulin	FMOD	Collagen fibril organization	0.96	↑
ANXA1_RABIT	Annexin A1	ANXA1	Calcium ion binding	0.95	↓
G1SWV4_RABIT	Thrombospondin 4	THBS4	Calcium ion binding	0.92	↓
PPIA_RABIT	Peptidyl-prolyl cis-trans isomerase A	PPIA	Protein folding	0.91	↑
G1T4A5_RABIT	Collagen alpha-1(I) chain	COL1A1	Extracellular matrix structural constituent	0.90	↑
G1SCK5_RABIT	Serpin family F member 1	SERPINF1	Serine-type endopeptidase inhibitor activity	0.90	↑
HBA_RABIT	Hemoglobin subunit alpha-1/2	Hba1 *	Heme binding	0.89	↑
SODE_RABIT	Extracellular superoxide dismutase [Cu-Zn]	SOD3	Heparin binding	0.89	↑
G1SD89_RABIT	Collagen type VI alpha 2 chain	COL6A2	Cell-binding protein	0.85	↑
G1U6M8_RABIT	Collagen type VI alpha 1 chain	COL6A1	Cell-binding protein	0.81	↑
G1T994_RABIT	Collagen alpha-1(XII) chain	COL12A1	Extracellular matrix structural constituent conferring tensile strength	0.80	↑

## Supplementary Materials

The following are available online at https://www.mdpi.com/2227-9059/8/10/408/s1, Table S1: Mass spectrometry files.
